# Caught in the act

**DOI:** 10.7554/eLife.01127

**Published:** 2013-08-08

**Authors:** Hermann-Josef Meyer, Michael Rape

**Affiliations:** 1 **Hermann-Josef Meyer** is in the Department of Molecular and Cell Biology, University of California, Berkeley, Berkeley, United Stateshermannmeyer@berkeley.edu; 2 **Michael Rape** is in the Department of Molecular and Cell Biology, University of California, Berkeley, Berkeley, United Statesmrape@berkeley.edu

**Keywords:** ubiquitin, HECT, E3 ligase, E2 conjugating enzyme, NEDD4, Rsp5, *S. cerevisiae*

## Abstract

The crystal structure of a HECT E3 enzyme has been captured as it transfers ubiquitin to a target protein, revealing the dramatic changes in shape that enable it to modify particular residues in its targets.

**Related research article** Kamadurai HB, Qiu Y, Deng A, Harrison JS, MacDonald C, Actis M, Rodrigues P, Miller DJ, Souphron J, Lewis SM, Kurinov I, Fujii N, Hammel M, Piper R, Kuhlman B, Schulman BA. 2013. Mechanism of ubiquitin ligation and lysine prioritization by a HECT E3. *eLife* **2**:e00828. doi: 10.7554/eLife.00828

**Image** The HECT E3 enzyme is part of a three-enzyme relay that transfers ubiquitin (yellow) to its target proteins

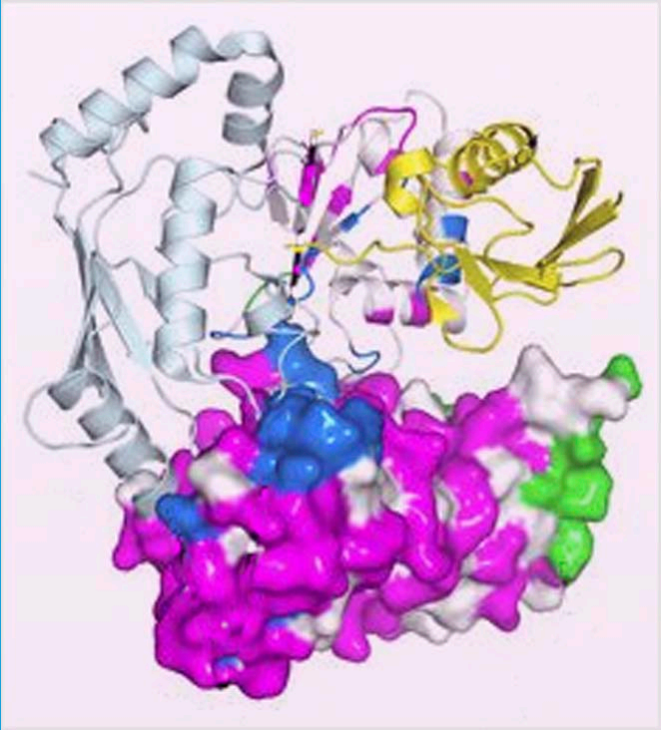

Most processes in the cell must be strictly controlled to ensure that they occur only when and where they should. The proteins that carry out these processes are often regulated by modifying them so as to turn them on or off, or to influence their interactions with other proteins. One such modification is ubiquitin, a small protein that can be linked to target proteins to regulate their behaviour. A trio of enzymes, called E1, E2 and E3, are responsible for activating a ubiquitin molecule and then attaching it to a target protein in a process known as ubiquitylation (Figure 1).

**Figure 1.**
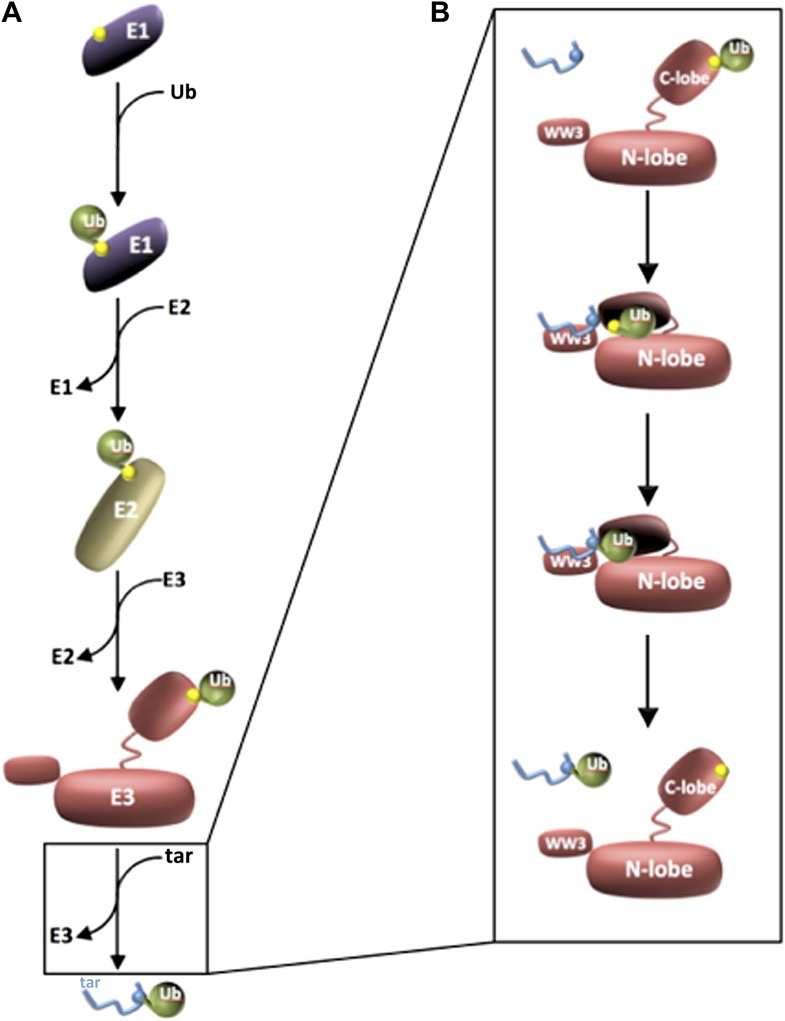
Three enzymes are needed to activate ubiquitin and attach it to a target protein. (**A**) Ubiquitin (green) is activated by an E1 enzyme and becomes covalently linked to a cysteine residue in the active site of the enzyme (all active sites are marked in yellow). The ubiquitin is then transferred to a cysteine residue in the active site of an E2 enzyme, which delivers it to a cysteine residue in the active site of the E3 enzyme’s HECT domain. In each case ubiquitin is attached to the enzyme by a thioester bond. The E3 then catalyses the transfer of the ubiquitin to a target protein (blue; labelled ‘tar’). (**B**) Kamadurai et al. have studied the last stage of this process in detail for an E3 enzyme called Rsp5. The HECT domain of the E3 enzyme is composed of an N-lobe (which binds the E2 enzyme) and a C-lobe, which contains the active site (yellow) and also a non-covalent binding site that stabilizes the ubiquitin. The target protein is detected by Rsp5 through an additional domain called the WW3 domain (top panel). The C-lobe then undergoes a major rearrangement that leads to the formation of a composite active site comprising residues of both N- and C-lobes. This rearrangement also places the active site in proximity to the target protein (second panel), which helps the enzyme to select specific lysine residues in the target protein for modification (third panel). The ubiquitylated target protein is then released (bottom panel).

By bringing together a target protein with activated ubiquitin, the E3 enzyme catalyses the transfer of this ubiquitin to a specific amino acid residue within the target (a lysine) (Komander and Rape, 2012). However, the details of this process are not well understood. Now, in *eLife*, a group led by Brenda Schulman, of the St Jude Children’s Research Hospital in Memphis, and Brian Kuhlman, of the University of North Carolina at Chapel Hill, report that they have used structural and biochemical assays to explore how an essential E3 enzyme modifies specific residues in target proteins (Kamadurai et al., 2013).

Schulman, Kuhlman and co-workers—including Hari Kamadurai as first author—have captured the moment when Rsp5, an E3 enzyme that is essential in yeast, transfers a ubiquitin molecule to a lysine residue within a target protein called Sna3. E3s comprise a large class of enzymes, with more than 600 members in human cells; Rsp5 belongs to the HECT family of E3 enzymes, which can regulate essential transcription factors, intracellular trafficking, or the cellular response to stress (Rotin and Kumar, 2009). Accordingly, mutations in genes encoding HECT E3s can lead to several diseases, among them inflammatory syndromes or hypertension.

The catalytic portion of the HECT E3 enzyme, which adds ubiquitin to the target protein, is called the HECT domain; this region is composed of two lobes—the N-lobe and the C-lobe—that are connected by a flexible linker. Intriguingly, previous research suggested that these lobes might adopt a variety of positions with respect to each other during the attachment of ubiquitin to the target protein. Moreover, it appeared that there might be a considerable distance between the relevant lysine in the target protein and the active (catalytic) site of the HECT domain (Verdecia et al., 2003; Kamadurai et al., 2009). These details have frustrated attempts to model how HECT E3s might transfer ubiquitin to a target protein and, in particular, to a specific residue in that target.

The C-lobe of the HECT domain contains a cysteine residue, which is charged with ubiquitin through a thioester bond to create an essential catalytic intermediate also found in a few other classes of E3 enzymes (Wenzel et al., 2011). The C-lobe also provides a site for non-covalent interaction with ubiquitin: this stabilizes the covalent bond between ubiquitin and the E3 enzyme, and it also helps to transfer ubiquitin to the target protein (Kamadurai et al., 2009; Maspero et al., 2013). The N-lobe possesses a key binding site for E2 enzymes, which load ubiquitin onto the active site of the HECT E3 (Figure 1) (Kamadurai et al., 2009). Using a bivalent crosslinker attached to a specific site in a target peptide, Kamadurai et al. revealed that the transfer of ubiquitin from the E3 enzyme to the target protein coincides with a dramatic re-organization of the HECT domain, with the C-lobe swinging through an angle of ∼130° to place the catalytic centre next to the target lysine.

In addition to closing the gap between the active site and the modified lysine, this rearrangement produces a large region of interaction between the two lobes of the HECT domain that serves important functions. First, it clamps down on the flexible C-terminal tail of ubiquitin, thereby orienting the thioester bond for nucleophilic attack by the lysine on the target protein. It also leads to formation of a composite active site that contains both the catalytic cysteine in the C-lobe and an acidic residue of the N-lobe that might orient or deprotonate the substrate lysine. Lastly, the arrangement of the catalytically engaged HECT domain restricts the flexibility of the active site relative to the portion of the E3 that associates with the target protein, a feature that enables the enzyme to prioritize the lysine that is modified in vivo. Other E3 enzymes have recently been seen to tether the activated ubiquitin during catalysis (Saha et al., 2011; Wickliffe et al., 2011; Dou et al., 2012; Plechanovová et al., 2012; Pruneda et al., 2012), and the orientation of the catalytic site relative to motifs that can interact with target proteins might also help to determine how E3s with different catalytic domains select lysines to modify on target proteins (Wu et al., 2003; Williamson et al., 2011). The present findings therefore raise the exciting hypothesis that very different classes of E3 enzymes evolved similar mechanisms to modify target proteins rapidly and specifically.

In addition to adding a single ubiquitin to a lysine within a target protein, HECT E3s can also assemble polymeric ubiquitin chains that are connected through one of a few dedicated lysines of ubiquitin (lysines 29, 48 or 63) (Rotin and Kumar, 2009). Previous studies have found that the C-lobe of the HECT domain can determine which one of these lysines is used in chain formation (Kim and Huibregtse, 2009), while Kamadurai et al. point to a requirement for N-lobe residues in coordinating this process. It will be interesting to see if HECT E3s can also be ‘caught in the act’ of assembling a chain, as this could reveal how multiple interactions contributed by several domains of the enzyme establish catalytic efficiency and specificity.

Collectively, these new insights underscore the importance of structural rearrangements for catalysis, a phenomenon that might be referred to as macromolecular juggling (Lorenz et al., 2013). Pessimists might view these idiosyncrasies of ubiquitylation reactions as posing challenges to the identification of small molecules that can target disease-relevant E3s. Optimists, however, might see the architectural changes that occur during ubiquitylation as providing opportunities for drug discovery. Hopefully, innovative approaches such as those documented in this study will help to harness the potential of ubiquitylation enzymes for therapeutic purposes.
